# Validity and Reliability of a New Specific Parkour Test: Physiological and Performance Responses

**DOI:** 10.3389/fphys.2019.01362

**Published:** 2019-10-30

**Authors:** Johnny Padulo, Luca Paolo Ardigò, Massimo Bianco, Drazen Cular, Dejan Madic, Branko Markoski, Wissem Dhahbi

**Affiliations:** ^1^Dipartimento di Scienze Biomediche per la Salute, Università degli Studi di Milano, Milan, Italy; ^2^Sport Performance Lab, University of Split, Split, Croatia; ^3^Department of Psychology, University eCampus, Novedrate, Italy; ^4^School of Exercise and Sport Science, Department of Neurosciences, Biomedicine and Movement Sciences, University of Verona, Verona, Italy; ^5^Faculty of Kinesiology, University of Split, Split, Croatia; ^6^Faculty of Sport and Physical Education, University of Novi Sad, Novi Sad, Serbia; ^7^Technical Faculty “Mihajlo Pupin”, University of Novi Sad, Zrenjanin, Serbia; ^8^Sport Science Program, College of Arts and Sciences, Qatar University, Doha, Qatar

**Keywords:** field test, muscle strength, physiological demands, sport science, testing

## Abstract

Main aim of this study was examining validity and reliability of using a new specific Parkour repeated sprint ability test (SPRSA) for assessing repeated sprint ability while facing obstacles and establishing between-day reliability and sensitivity of SPRSA related to its physiological and performance responses. Thirteen high-level traceurs (three females) performed in random order and twice eight tests for assessing a total of 23 variables: SPRSA (a typical maximal-speed shuttle run interspersed with four Parkour competition-common fundamentals) and seven established fitness tests, core stability, hand-grip, vertical-jump, long-jump, pull-up, 300-m shuttle run (as a field test for anaerobic capacity), and Leger test. Except for muscular elasticity index of vertical jump test (intra-class Correlation Coefficient model 3,1 [ICC_3_,_1_] = 0.54 [*fair*]), fitness tests’ ICC_3_,_1_s resulted *excellent* (ICC_3_,_1_: 0.93–1.00). SPRSA total time and time of its fastest sprint (SPRSA peak time) were significantly correlated with the majority of core stability (*r*: −0.79 to 0.59; *P* < 0.01–0.05), jumping (*r*: −0.78 to 0.67; *P* < 0.01–0.05), pull-up tests (*r*: −0.86; *P* < 0.01), 300-m shuttle run test total time (*r*: 0.77–0.82; *P* < 0.01), and Leger test-estimated VO_2_ max (*r*: −0.78; *P* < 0.01). Principal component analysis (PCA) of the 23 variables led to extraction of four significant components (each due to different variables’ combinations), which explained 90.2% of 23 variables’ total variance. SPRSA (i.e., total and peak time) showed high reliability (ICC_3_,_1_: 0.991–0.998 and standard-error-of-measurement %: 0.07–0.32). Finally, SPRSA showed high sensitivity (smallest-worthwhile-change %: 0.29–0.68). Considering its excellent logical and strong ecological validity, SPRSA may serve as a valid specific field test for Parkour sport. In addition, thanks to its high reliability and sensitivity, this test is suitable for monitoring, evaluating, and programming training processes for Parkour practitioners in repeated sprint ability involving crossing obstacles.

## Introduction

Parkour is a relatively new individual sport in which athletes try to negotiate different obstacles by swinging, running, jumping, and climbing (Coolkens et al., 2018). Parkour athletes specialize in going through intricate urban environments, which are based on three dimensions. Meanwhile, ground contact should be avoided as much as possible (Strafford et al., 2018). Over such movements, limbs are involved in a wide range of joint positions (Halsey et al., 2017). Athletes’ interactions with surrounding environment should be cognition, perception, and action-based as they become skilled at performing similar or different movements respecting the properties of the different obstacles and surfaces (Strafford et al., 2018). Parkour is an acrobatic sport, in which practitioners exploit movement skills such as running, climbing, jumping, bi- or uni-pedal landing, hanging, vaulting, balancing, stepping, hurdling, quadrupedal movement, and rolling. Likewise, perceptual/control abilities (coordination, timing, balance, agility, spatial awareness, and muscular strength) should be developed so that athletes can effectively cope with environmental features such as gaps, obstacles, surfaces, and inclines (Aggerholm and Højbjerre Larsen, 2017). Carrying out Parkour activities necessitates athletes’ involvement in calculating distances, gap sizes, and surface properties. It also familiarizes them with environmental features through cognitive skills such as perception, concentration, creativity, and problem solving (Strafford et al., 2018). Parkour has been considered as a distinctive kind of activity thanks to its limited amount of classical coaching. In fact, skills are not acquired through classical ways of learning like rigid instructions delivered by coaches, but they are rather experience, observation, and exploration-based (Aggerholm and Højbjerre Larsen, 2017).

Recently, Parkour has been growing more and has become more popular. Therefore, hundreds of youngsters are practicing it worldwide thanks to its beneficial effects on physical fitness (Taylor et al., 2011; Grosprêtre and Lepers, 2016), mental health, and social learning (Grosprêtre and Lepers, 2016; Grosprêtre et al., 2018). Furthermore, Parkour tremendously contributes to team-game talent development as athletes are compelled to face challenging obstacles with different textures, surfaces, inclinations, areas, sizes, and angles. Fore and foremost, they have to adjust their movement behaviors so as to fit the changing environmental backgrounds (Strafford et al., 2018).

Plyometric and eccentric exercises are the main components of Parkour. They are frequently carried out with relevant mechanical stress especially in the case of high drop jumps (Miller and Demoiny, 2008). For Parkour athletes, the entire world becomes a suitable playground within an urban environment. By making use only of their physical skills and talents, Parkour practitioners (or “traceurs”) aim to get from one point to another in a complex environment full of obstacles, without assistive equipment and in the fastest and most efficient way possible. They should learn to use common objects like trees, rails, benches, and walls as exercise equipment to perform a wide range of movements including long and standing jumps, drop jumps, fast climbing, among others (Miller and Demoiny, 2008). In a recent study, traceurs proved to be more successful than other power athletes (e.g., gymnastics and field athletes) in exercises such as specific jumps. They showed more remarkable eccentric forces as well (Grosprêtre and Lepers, 2016). Therefore, traceurs represent a new type of athlete able to effectively combine plyometric exercise with eccentric strength. Traceurs intend to attain perfection through developing physical ability and spatial awareness using the “tic tac” technique. Such technique consists in moving toward obstacles and taking off with a change of direction. The athlete here must have clear objectives while dealing with obstacles or drawing on perceptual variables. For example, he/she has to calculate the time needed before having any contact with the object to anticipate and thus adjust the following movement phase (Strafford et al., 2018). So far, very few studies have considered how Parkour could cause force and jump skills to develop. Most of them have focused on sociological and psychological sides (Taylor et al., 2011). As far as physiological data are concerned, nothing has really been highlighted but the injuries resulting from falls while Parkour activities are performed (Miller and Demoiny, 2008).

Data deficiency in the literature about the physical characteristics of traceurs is quite evident. Yet, that might help to include one further tool to measure coaching and testing in Parkour and thus to highlight traceurs’ degree of psychomotor skill and fitness development or degradation (Kim et al., 2014). If Parkour skills and fitness have to be assessed over real performance, evaluation standards need to be especially established accordingly. This study’s main objective was to examine the scientific legitimacy of using the new specific Parkour repeated sprint ability test (SPRSA) for assessing repeated sprint ability while facing obstacles. Given that all Parkour events call for restricted-time (*Alive After 5*, *minimum* three 3-min bouts; *Best Trick*, four 1-min bouts; *Style*, one 3-min bout; *Pairs Battle*, five 30-s bouts) or timed trials (*Speed*; *Pairs Speed*; *Dueling Speed*; and *Relay*)^1^, Parkour intermittent high-intensity activity strongly resembles repeated sprint ability test paradigm in any case. The fact that Parkour short runs are interspersed in all its specific explosive fundamentals (e.g., swinging, running, jumping, and climbing)^2^ makes it very similar to a repeated sprint ability test with all its (re)starts, changes of direction, and stops. Moreover, Parkour does require a high-level in all the above-mentioned physical abilities, which some commonly used tests correspond to. Therefore, we planned to administer such tests to participants, as well.

Looking for correlation between SPRSA and some fitness tests performances seems more solid in terms of face validity, but its disadvantage is that it is based on many variables, which complicate interpretation of results. As such, this method may fall into redundant use of performance indices. Among best solutions to reduce these disadvantages there is principal component analysis (PCA), a statistical approach used to model and highlight selected data (Zalleg et al., 2018). Main advantage of PCA is that it facilitates illustration of good models and reduction in variables and number of dimensions, with *minimum* loss of information (Milovanović and Popović, 2012). Therefore, PCA method was selected for physical and physiological characterization of the new test used in this investigation. Secondary aim was to establish the between-day reliability, sensitivity and minimal detectable change of SPRSA and between-day reliability of the fitness tests.

## Materials and Methods

### Participants

Using G^∗^Power software (Bonn FRG, Bonn University, Department of Psychology), we found out that 11 subjects were needed to achieve a statistical power of 80% to detect a *small* effect (*d* = 0.182) regarding SPRSA main variables taken into consideration (viz. total and peak time and percent decrement) with a level of significance of 5% and independently of sex. Therefore, we recruited 13 voluntary traceurs (3 women) to ensure that no data would be lost. All anthropometric data (age, body height and mass, body mass index, and total percentage of body fat) can be found in Table 1. Inclusion *criteria* for participation in this study were: a *minimum* weekly training frequency of three sessions or a total time of 120 min of Parkour activities *per* week, more than one year of Parkour training experience, and the absence of any physical impediment like injury or pain that might hamper participants from making maximal effort at some stage of a Parkour test. Giving a detailed report of the study’s procedures and rules encouraged participants or their parents – in case of minor age – to provide a formal written consent to take part in the study. The protocol conformed to internationally accepted policy statements regarding the use of human participants in accordance with the Declaration of Helsinki and was approved by the local university’s ethics committee.

**TABLE 1:** Descriptive statistical indicators of morphological variables (*n* = 13, 10 men and 3 women).

**Variable**	**Average**	**Minimum**	**Maximum**	**Standard deviation**	**CI 95%**
Age (years)	19.08	16	22	3.68	16.85–21.30
TE (years)	3.31	1	9	2.09	2.04–4.57
TW (h)	7.08	2	14	3.73	4.82–9.33
BH (cm)	170.15	152	184	10.02	164.10–176.21
BM (kg)	64.43	38.20	98.60	14.28	55.80–73.07
BMI (kg m^–2^)	21.97	16.50	29.10	3.32	19.96–23.98
FATP (%)	14.58	5.90	27.00	7.25	10.20–18.97

*TE = training experience; TW = training week; BH = body height; BM = body mass; BMI = body mass index; FATP = total percentage of body fat; CI = confidence interval.*

### Protocol

A cohort-based, randomized, repeated measures study design was used. Experimental protocol consisted in performing SPRSA and some fitness tests. Only 1 week before first tests, participants were summoned – over a single session – to get familiarized with study experimental protocol. First session of testing was dedicated to all assessment tests but SPRSA, 300-m shuttle run, and Leger test, and namely core stability battery test, hand-grip (both sides), vertical jumps, long jumps, and pull-up. The protocol consisted in performing the tests in random order, with at least 10-min recoveries between each test and the next one to avoid fatigue influence on tests’ outcomes. SPRSA, 300-m shuttle run, and Leger test were administered on three, following separate days. All (15) assessment tests were performed twice with 1 week in-between.

Results about the Parkour tests were gathered at about the same timing in both sessions (from 09:00 to 11:00 a.m.) so that any influence on circadian variations on performance could be avoided (Ammar et al., 2015). Participants were invited not to change their eating or sleeping habits. Besides, they were asked not to have a heavy meal 3 h before each session at least. Fore and foremost, they were advised to avoid doing any exhausting activity during the 24 h before the test. During the tests, participants were verbally stimulated by the experimenter in order to attain *maximum* effort. To ensure the same testing conditions, the same raters tested all participants. The test was performed using a specially designed measuring board out of doors in a field (measurements were taken every 30 min during the experiment): temperature 20 ± 0.5°C and humidity 50 ± 10%, monitored by means of a digital environmental station (Vaisala Oyj, Helsinki, Finland) during test and retest sessions.

#### Specific Parkour Repeated Sprint Ability

**FIGURE 1:**
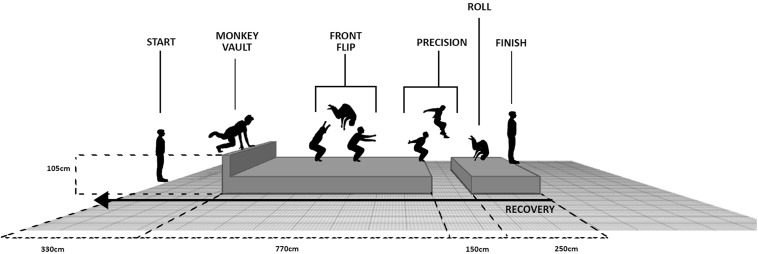
New specific Parkour repeated sprint test with four competition-common fundamentals over forward leg (backward leg = 15-s recovery jogging).

SPRSA was a 15-m, 10-time, maximal-speed shuttle run, with a 15-s recovery jogging way back (Figure 1). Over each shuttle, traceurs had to perform four competition-common fundamentals in the following order: monkey vault, front flip, precision, and roll. Photocell gates (Brower Timing System, Salt Lake City, UT, United States; accuracy of 0.01 s) were used to time each run for total time, and time of its fastest sprint (peak time) and to calculate percent decrement. Rates of perceived physical exertion (RPE, as a value of a 6–20 scale; Borg, 1982) were indicated by traceurs after each shuttle run.

#### Core Stability

Core stability was assessed by means of five common exercises: *Face plank*, *Left side plank*, *Right side plank*, *Hamstring*, and *Quadriceps* (Dello Iacono et al., 2016). *Maximum* time each traceur was able to go on with each exercise was measured with a stopwatch. In addition, an overall variable, *Total points*, was calculated considering all exercise times as from 0 s to 1 min 30 s = 1 point, from 1 min 30.01 s to 3 min = 2 points, from 3 min 0.01 s to 4 min 30 s = 3 points, and from 4 min 30.01 s to 6 min 30 s = 4 points.

#### Hand-Grip

Hand-grip, as strength test, was carried out following the protocol that was exposed by España-Romero et al. (2019). Both hands (i.e., left and right) were evaluated. The experimenter chose randomly the hand that he/she would test first. The Takei Hand Grip Dynamometer (Takei A5401 Digital Hand Grip Dynamometer; error 0.001 g) was used, a digital tool with an adjustable grip span (Balogun and Onigbinde, 1991).

#### Vertical Jump

For squat-jump (SJ), experimenters asked participants to initiate from an upright standing position and to keep their hands on their hips (Laffaye et al., 2016). Experimenters also instructed participants to keep their knees flexed in a position assigned (∼90°) for a 3-s count (Čular et al., 2018b). Subsequently experimenters asked participants to jump as high as possible provided they did not make any countermovement (Padulo et al., 2013). For countermovement jump (CMJ), subjects held their hands on their knees – without moving them – in an upright standing position. After that, experimenters invited participants to flex down their knees (∼90°) in a fast and swift way and then jump as high as possible in the following concentric phase. Experimenters did not consider incorrect jumps, but, on the contrary, they urged participants to retry. To measure vertical ground reaction force during jumping, a force platform (Quattro-Jump 9290AD, Kistler, Winterthur, Switzerland) was tightly placed on the ground. A personal computer with manufacturer’s software (QJ 1.0.9.2, Kistler, Winterthur, Switzerland) was connected to force platform providing vertical jump assessment. In addition to specific SJ and CMJ assessments, elastic contribution due to CMJ’s countermovement was calculated as the muscular elasticity variable, i.e., (CMJ height–SJ height)/CMJ height (%) (Komi and Bosco, 1978).

#### Long Jump

Long jump performance was assessed by administering traceurs two tests: from standing (*Standing long jump*) and after a 15-m run-up (*Leaping long jump 15 m*). Long jump distances were measured with a measuring tape. In addition, an overall variable, *Total points*, was calculated simply as *Standing long jump distance* + *Leaping long jump 15 m distance* (Padulo et al., 2014).

#### Pull-Up

For the pull-up test, participants hung from a horizontal bar (5-cm diameter), with hands between one and one-and-a-half shoulder-widths apart from each other, with prone grip (i.e., with palms turned away from face), and arms fully extended. To execute a successful pull-up, participants had to have their chins clear the bar (i.e., they had to lift their chins above the bar). These exercises are called chin-ups and they are effective for building bigger arms. Unfortunately, chin-ups are spoiled if participants try to swing their bodies, neglect to stretch their arms fully (i.e., absence of full arm extension), or lift their chins (i.e., neck extension; Dhahbi et al., 2018).

#### 300-m Shuttle Run

As a field test for anaerobic capacity, the 300-m shuttle run test was used, consisting of a 20-m, 15-time, maximal-speed, and continuous shuttle run (Moore and Murphy, 2003). 300-m shuttle run test times were measured with a photocell gate (Brower Timing System, Salt Lake City, UT, United States; accuracy of 0.01 s; Dhahbi et al., 2018).

#### Leger Test

*Maximum* oxygen consumption (VO_2_ max) was estimated with the maximal multistage 20-m shuttle run Leger test [standard error of measurement (SEM) 4.7 ml kg^–1^ min^–1^; Léger et al., 1988].

### Statistical Analysis

SPSS version 23.0 for Windows (SPSS, Inc., Chicago, IL, United States) was used to perform data analyses. After verifying the normality of distributions with the Kolmogorov–Smirnov method, it were calculated means and standard deviations. Pearson’s correlation coefficient estimations were calculated to assess strength of relationships between SPRSA and fitness tests variables. Moreover, PCA was performed so that the main component summarizing the 23 considered variables was found. At this stage, the procedure illustrated by Kollias et al. (2001) was used. Quite an important amount of principal components in the pattern matrix extracted by PCA was selected with an eigenvalue higher than 1 (Kaiser *criterion*). Original matrix was rotated to extract the appropriate variables using a normalized varimax (*maximum* variation) rotation. Relative reliability of SPRSA and fitness tests was assessed by calculating their Intra-class Correlation Coefficient model 3,1 (ICC_3_,_1_). Furthermore, absolute reliability of SPRSA was expressed in terms of its SEM and coefficients of variation (CV). In order to assess test sensitivity, it was resorted to weighing smallest worthwhile change (SWC) against SEM, focusing on the thresholds proposed by Liow and Hopkins (2003). Minimal detectable change at 95% confidence interval (MDC_95_) was also calculated for SPRSA variables. Heteroscedasticity was investigated. Significance for all the statistical tests was set at *P* ≤ 0.05.

## Results

Results showed data were normally distributed (Kolmogorov-Smirnov *P* = 0.06–0.88). Except for muscular elasticity index of vertical jump test (ICC_1_,_3_ = 0.54 [*fair*]), fitness tests’ ICC_3_,_1_s resulted *excellent* (ICC_3_,_1_: 0.93–1.00). Descriptive SPRSA and fitness tests performances collected are shown in Table 2. It is noteworthy that lack of sex effect was confirmed regarding all our new SPRSA variables (Supplementary Material).

**TABLE 2:** Descriptive statistical indicators and Intra-class Correlation Coefficient reliability indexes (*n* = 13, 10 men and 3 women).

**Tests**	**Variable**	**Average**	**Minimum**	**Maximum**	**SD**	**CI 95%**	**ICC_3_,_1_**
Core stability	Face plank (s)	210.05	97.47	342.34	78.96	162.34–257.77	1.00
	Left side plank (s)	102.38	56.70	157.21	35.82	80.74–124.02	0.99
	Right side plank (s)	103.02	60.13	163.58	37.57	80.31–125.72	0.99
	Hamstring (s)	163.60	55.46	304.54	73.59	119.13–208.07	1.00
	Quadriceps (s)	132.55	65.34	190.11	40.00	108.38–156.72	1.00
	Total points (score)	10.38	6.00	14.00	2.79	8.70–12.07	1.00
Hand-grip	Dominant (kg)	43.38	20.70	75.30	14.75	34.47–52.30	0.99
	No dominant (kg)	40.81	22.40	71.10	13.14	32.87–48.75	0.99
Vertical jump	SJ height (cm)	35.60	22.67	42.97	6.19	31.86–39.34	1.00
	SJ net impulse (Ns)	171.15	96.12	272.29	46.54	143.03–199.27	1.00
	CMJ height (cm)	38.29	24.39	46.67	6.93	34.10–42.48	0.98
	CMJ net impulse (Ns)	177.54	99.30	282.44	48.84	148.03–207.06	0.93
	Muscular elasticity (%)	7.87	4.35	12.27	2.20	−128.31 to 58.37	0.54
Long jump	Standing long jump (cm)	198.46	149.00	239.00	28.62	181.17–215.76	0.99
	Leaping long jump 15 m (cm)	355.77	250.00	407.00	48.90	326.22–385.32	1.00
	Total points (cm)	554.23	399.00	636.00	75.85	508.40–600.07	1.00
Pull-up	Pull-up (score)	11.00	1.00	20.00	5.18	7.87–14.13	0.98
300-m shuttle run test	Total time (s)	92.79	83.53	135.06	14.08	84.28–101.30	0.98
Leger test	VO_2_ max (ml kg^–1^ min^–1^)	45.37	40.25	50.75	2.72	43.72–47.01	1.00
SPRSA	Total time (s)	85.26	63.30	122.57	22.06	71.93–98.59	1.00
	Peak time (s)	6.75	4.80	10.20	1.81	5.65–7.84	0.99
	Percent decrement (%)	26.63	20.07	36.00	5.49	23.31–29.95	0.75
	RPE (score)	16.38	13.00	18.00	1.85	15.27–17.50	1.00

*SD = standard deviation; SPRSA = specific Parkour repeated sprint ability test; CMJ = countermovement jump; SJ = squat jump; RPE = rating of perceived exertion; ICC_3,1_ = Intra-class Correlation Coefficient model 3,1.*

Pairwise analysis of SPRSA total time, peak time, and percent decrement indices revealed no significant between-days difference (*P* = 0.053–0.217). Moreover, total time and peak time assessments showed an *excellent* reliability (ICC_3_,_1_ s > 0.99, CV: 1.47–3.39%, and SEM: 0.07–0.32%). On the other hand, SPRSA assessments showed a good sensitivity, given that SEM values were smaller than SWC ones (SWC = 0.29 and 0.68% for total time and peak time, respectively). In addition, MDC_95_ for total time and peak time were small (≤0.16 s, Table 3). On the other hand, percent decrement showed poor absolute reliability (CV = 11.79% and SEM = 5.85%) and marginal sensitivity (SEM = 5.85% > SWC = 2.37% and MDC_95_ = 16.21%). Heteroscedasticity coefficients for total time, peak time, and percent decrement variables were all “strong” and significant (*r*: 0.61–0.71; *P*: 0.007–0.028).

**TABLE 3:** Relative and absolute reliability variables and minimal detectable change at 95% confidence interval of the specific Parkour repeated sprint ability test.

**Variable**	**Mean ± SD**		**ICC_3,1_ (95%CI)**	**CV%**	**SEM (%)**	**SWC (%)**	**MDC_95_ (%)**
						
	**Session 1**	**Session 2**					
Total time (s)	85.26 ± 22.06	85.73 ± 21.30	0.998 (0.995–0.999)	1.47	0.06 (0.07)	0.25 (0.29)	0.16 (0.18)
Peak time (s)	6.75 ± 1.81	6.66 ± 1.64	0.991 (0.972–0.997)	3.39	0.02 (0.32)	0.05 (0.68)	0.06 (0.89)
Percent decrement (%)	26.63 ± 5.49	28.57 ± 3.60	0.754 (0.370–0.918)	11.79	1.61 (5.85)	0.65 (2.37)	4.47 (16.21)
RPE (score)	16.38 ± 1.85	16.38 ± 1.85	1.00 (1.00–1.00)	0.00	0.00 (0.00)	0.00 (0.00)	0.00 (0.00)

*RPE = rating of perceived exertion; ICC_3,1_ = Intra-class Correlation Coefficient model 3,1; CV = coefficient of variation; SEM = standard error of measurement; SWC = smallest worthwhile change; MDC_95_ = minimal detectable change at 95% confidence interval.*

**FIGURE 2:**
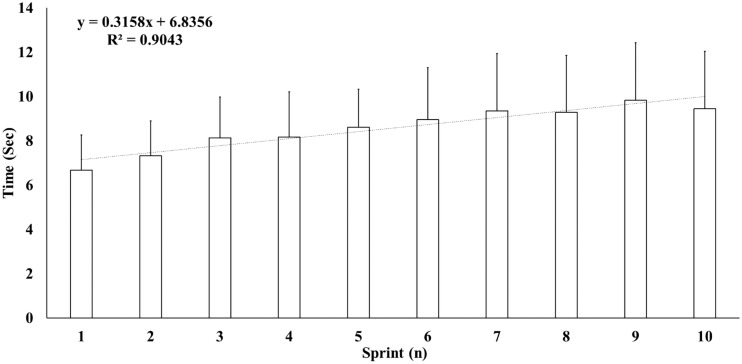
New specific Parkour repeated sprint test sprint times (means and standard deviations).

Total time, peak time, and RPE of SPRSA (Figure 2) were significantly correlated with the majority of core stability tests: *Left side plank*, *Right side plank*, *Quadriceps*, and *Total points* (*r*: −0.79 [*strong*]–0.59 [*moderate*]; *P*: 0.001–0.035). Furthermore, total time was significantly correlated with jump and pull-up tests: SJ height, CMJ height, standing long jump, *Leaping long jump 15 m*, and *Total points* (*r*: −0.87 [*very strong*]–0.57 [*moderate*]; *P*: 0.002–0.041). In addition, total time and peak time of SPRSA correlated with 300-m shuttle run test total time (*r* = 0.78 [*very strong*]; *P* = 0.002 and *r* = 0.82 [*very strong*]; *P* = 0.001, respectively) and with VO_2_ max estimated from Leger test (*r* = 0.78 [*very strong*] and *r* = 0.78 [*very strong*], respectively; *P* = 0.002; Table 4).

**TABLE 4:** Correlation matrix for the entire group.

		**SPRSA**			
		
**Tests**		**Total time**	**Peak time**	**Percent decrement**	**RPE**
Core stability	Face plank	–0.345	–0.371	0.237	–0.484
	Left side plank	–0.789^∗∗^	–0.793^∗∗^	0.155	–0.734^∗∗^
	Right side plank	–0.744^∗∗^	–0.742^∗∗^	0.115	–0.729^∗∗^
	Hamstring	–0.206	–0.150	–0.172	–0.320
	Quadriceps	−0.618^∗^	−0.588^∗^	–0.023	–0.704^∗∗^
	Total points	−0.638^∗^	−0.611^∗^	–0.015	−0.629^∗^
Hand-grip	Dominant	–0.137	–0.115	–0.105	–0.138
	No dominant	–0.117	–0.093	–0.121	–0.113
Vertical jump	SJ height	–0.724^∗∗^	–0.758^∗∗^	0.202	–0.514
	SJ net impulse	–0.228	–0.239	0.036	–0.187
	CMJ height	–0.776^∗∗^	–0.785^∗∗^	0.083	−0.571^∗^
	CMJ net impulse	–0.252	–0.256	–0.001	–0.213
	Muscular elasticity	–0.346	–0.231	–0.515	–0.265
Long jump	Standing long jump	−0.674^∗^	–0.692^∗∗^	0.161	−0.647^∗^
	Leaping long jump 15 m	–0.775^∗∗^	–0.784^∗∗^	0.078	−0.572^∗^
	Total points	–0.754^∗∗^	–0.767^∗∗^	0.111	−0.613^∗^
Pull-up	Pull-up	–0.858^∗∗^	–0.865^∗∗^	0.105	–0.783^∗∗^
300-m shuttle run test	Total time	0.775^∗∗^	0.820^∗∗^	–0.297	0.522
Leger test	VO_2_ max	–0.782^∗∗^	–0.780^∗∗^	0.044	−0.656^∗^

*SPRSA = specific Parkour repeated sprint ability test; CMJ = countermovement jump; SJ = squat jump; RPE = rating of perceived exertion. ^∗^Significant at 0.05 level (2-tailed). ^∗∗^Significant at 0.01 level (2-tailed).*

PCA of the 23 variables led to extraction of four significant components (each due to different variables’ combinations). The first rotated component explained 34.8% of the 23 variables’ total variance, whereas the second explained 25.3%, the third 22.2%, and the fourth 7.9% (Table 5). All principal components together explained 90.2% of the 23 variables’ total variance.

**TABLE 5:** Principal component analysis for specific Parkour repeated sprint ability and fitness tests including factor loadings, commonalities, eigenvalue for each variable, and percentage of variance for each rotated component. Factor loadings lower than 0.6 were not included in the table.

**Variables**	**Factor loadings**				**Commonalities**
		
	**1**	**2**	**3**	**4**	
SPRSA peak time	−0.914				0.953
SPRSA total time	−0.899				0.950
Pull-up	0.876				0.908
300-m shuttle run test total time	−0.870				0.815
VO_2_ max	0.863				0.778
CMJ height	0.783				0.916
SJ height	0.771				0.903
Leaping long jump 15 m	0.722				0.973
Long jump total points	0.700				0.981
SPRSA RPE	−0.691				0.733
SJ net impulse		0.968			0.978
CMJ net impulse		0.968			0.980
No dominant hand-grip force		0.961			0.951
Dominant hand-grip force		0.935			0.926
Standing long jump		0.675			0.891
Core stability total points			0.925		0.961
Hamstring			0.890		0.908
Face plank			0.886		0.894
Right side plank			0.832		0.968
Left side plank			0.809		0.969
Quadriceps			0.741		0.805
SPRSA percent decrement				−0.879	0.795
Muscular elasticity				0.826	0.805
Eigenvalue	8.00	5.82	5.10	1.82	
Percentage of variance	34.80	25.28	22.18	7.93	

*SPRSA = specific Parkour repeated sprint ability test; CMJ = counter-movement jump; SJ = squat jump; RPE = rating of perceived exertion.*

## Discussion

As far as we know, this study is really original since it is the first one ever to suggest an ecological assessment of validity, reliability, and sensitivity of a new specific Parkour skill test. Main aim of this research was to study the scientific legitimacy of using a new repeated sprint ability-inspired specific Parkour test (SPRSA) for assessing Parkour ability including its sub-ability of crossing obstacles. Further aim to establish SPRSA’s between-day reliability, sensitivity, and minimal detectable change and some fitness tests’ between-day reliability. Parkour’s testing is indeed a field where little is known (Grosprêtre and Lepers, 2016). Main findings are the significant relationships found between SPRSA’s total time, peak time, and RPE and the majority of core stability, jumping and pull-up, and 300-m shuttle run tests and VO_2_ max estimates. In addition, PCA revealed that the aforementioned 23 SPRSA and fitness tests variables resulted in the extraction of four significant components (each due to different variables’ combinations). Finally, SPRSA showed high reliability and sensitivity for assessing Parkour-specific repeated sprint ability especially regarding crossing obstacles. However, SPRSA’s absolute percent decrement showed poor absolute reliability and marginal sensitivity.

“A test has face validity or logical validity when it obviously measures the desired skill or ability” (Zalleg et al., 2018). As a test focused on basic athletic abilities and skills, SPRSA enables athletes to give proof of their real abilities and skills (Zalleg et al., 2018). In this study, what needed to be assessed was potential Parkour performance. To be more specific, we focused on a selected set of repeated sprints by crossing obstacles and involving upper and lower limbs like typical Parkour actions. Performance of SPRSA refers to jumping ability, sprint, and the explosive force produced by the upper limb as a whole. Consequently, we logically considered SPRSA as the most suitable test for Parkour performance so far.

Between-day reliability represents an important aspect of performance testing. Poor reliability might result in different scores for the examinee across two test sessions, which may lead to erroneous data interpretation (Currell and Jeukendrup, 2008; Čular et al., 2018a). Relative reliability, that shows maintenance of group position (rank order) of a test across two measures, can be measured by means of ICC (Currell and Jeukendrup, 2008). With ICC_3_,_1_s ranging from 0.75 [*good*] to 1.00 [*excellent*], SPRSA (e.g., its total time, peak time, and percent decrement) demonstrated a high relative reliability. For this reason, we really needed SEM examination that granted an absolute index of reliability (Weir, 2005) to confirm ICC results. SEM provides an estimate of measurement error. Moreover, if data are heteroscedastic, which is true for all variables of this study (*r*: 0.61–0.71; *P* < 0.05), CV analysis may be more useful than SEM in establishing absolute reliability (Currell and Jeukendrup, 2008). With homoscedastic data, SEM analysis is recommended (Atkinson and Nevill, 1998). Between-day CV values lower than 5% may be interpreted as good absolute reliability (Nevill and Atkinson, 1997). This investigation’s total time and peak time CVs were less than 3.5%. Total time and peak time SEMs were less than 1%, which can be considered good (<5%; Atkinson and Nevill, 1998), whereas percent decrement showed poor absolute reliability (SEMs and CVs > 5%). Furthermore, the likelihood that differences in SPRSA outcomes were paramount (i.e., SWC larger than the SEM) was equally assessed. As for total time and peak time, SEMs were smaller than SWCs (Table 3), indicating that measurements had “good” potential to detect real changes in performance outputs. In contrast, SWC for percent decrement (2.37%) was smaller than corresponding SEM (5.85%, Table 3). MDC_95_ values for total time, peak time, and percent decrement were 0.16 s, 0.06 s, and 4.47%, respectively. Thus, a change in total time, peak time, and percent decrement values exceeding 0.16 s, 0.06 s, and 4.47%, respectively, can be accepted as a true response (Atkinson and Nevill, 1998; Čular et al., 2018a). Furthermore, the likelihood that differences in SPRSA outcomes were paramount was equally assessed. For this reason, we can say that the suggested Parkour course and evaluation procedure seemed to be particularly effective to assess Parkour skill and fitness level over school or extracurricular physical education.

Parkour athletes show exceptional gymnastic and athletic skills that allow them to imitate the agility of arboreal monkeys (Halsey et al., 2017). They focus on building up new techniques that allow them to move through complex and three-dimensional urban environment limiting contact with the ground as much as possible (Halsey et al., 2017). Such techniques should involve limbs (arms and legs) in various joint positions and in both suspension and compression (Hunt et al., 1996). PCA can be a valuable tool for selectively limiting the closely interrelated physical requirement variables used to assess Parkour performance to a smaller number of variables to explain the same amount of data variance (Zalleg et al., 2018). Consequently, PCA can group together highly interrelated predictor variables at no risk of losing important information, eliminating the burden of dealing with too many variables (Kollias et al., 2001). PCA model used had four principal components and accounted for 90.18% of total variance of 23 variables selected as critical for assessing Parkour performance. SPRSA peak time loaded highly on first factor, with a factor loading of −0.91 (Table 5). SPRSA total time also showed a high negative factor loading (−0.90) on first component, indicating a high yet significant relationship with total time. The second rotated principal component was identified as vertical jump impulses and hand-grip forces (Table 5). SJ net impulse, CMJ net impulse, and both dominant and no dominant hand-grip force loaded highly on second component (commonalities ≥0.93). Urban Parkour performance frequently includes high-speed impacts against hard surfaces. For example, Parkour athletes usually jump down from different altitudes sometimes reported to be more than 20 feet high (6.1 m). They learn different landing techniques to manage two-footed landing impacts (precision) and roll (Croft and Bertram, 2017). Regarding physical characteristics, traceurs attain CMJ heights (38 cm) similar to both gymnasts’ and other power athletes’ (Grosprêtre and Lepers, 2016). Interestingly, traceurs attain greater SJ heights (36 cm) than both gymnasts and other power athletes (Grosprêtre and Lepers, 2016). Both traceurs’ and power athletes train a lot repeated jumps on hard surfaces such as concrete obstacles. This could justify their high achievements in CMJ and SJ performances. As regards to physical characteristics, current results show that traceurs show very important plyometric skills and exceptional upper-to-lower limb coordination, which are considered essential requirements for high-level long jump performance (Grosprêtre and Lepers, 2016). In addition, arboreal apes’ axial systems’ demands are particularly high, because high mobility and high grip forces are required for their body so that it could be maneuvered through the complex three-dimensional forest environment (Myatt et al., 2012). Such high demands result in forearm flexor muscles that are nearly four times larger than those in cursorial species like leopards or horses (Alexander et al., 1981). The third rotated principal component was identified as core stability (Table 5). Core stability total points loaded highly on third component (factor loading of 0.93). SPRSA percent decrement loaded highly negatively on fourth factor (factor loading of −0.88, Table 5). Muscular elasticity variable also showed a high but positive factor loading (0.83) on fourth component, indicating a high but not significant relationship with SPRSA percent decrement. In summary, 100% of SPRSA and fitness tests variables were well approximated by the principal components model as indicated by high communality scores, which ranged between 0.73 for SPRSA RPE and 0.98 for long jump *Total points*. Principal Components Analysis model displayed in this study revealed a great extrapolative performance in accounting for the SPRSA result.

Our sample of subjects may provide preliminary reference values for our test. Our new SPRSA results an effective and time-efficient test. As this research’s limitations, we acknowledge that a greater sample size and restricting it to only men or women would have made test’s reference values stronger.

## Conclusion

Specific Parkour repeated sprint ability test was originally designed to assess repeated sprint ability, especially regarding crossing obstacles, in Parkour practitioners. Significant relationships were found between SPRSA’s total time, peak time, and RPE and the majority of core stability, jumping and pull-up, 300-m shuttle run tests, and VO_2_ max estimates. In addition, a huge extrapolative physical fitness profile was revealed by the PCA model in traceurs for SPRSA. SPRSA also showed high reliability and sensitivity for assessing. All this indicates SPRSA has strong logical and ecological validity as a test of Parkour-specific repeated sprint ability, especially with regard to crossing obstacles.

## Data Availability Statement

The datasets generated for this study are available on request to the corresponding author.

## Ethics Statement

The studies involving human participants were reviewed and approved by the University of Novi Sad Ethics Committee. All subjects or their parents gave their written informed consent to participate in the study after receiving a thorough explanation of the study’s protocol.

## Author Contributions

All authors listed have made a substantial, direct and intellectual contribution to the work, and approved it for publication.

## Conflict of Interest

The authors declare that the research was conducted in the absence of any commercial or financial relationships that could be construed as a potential conflict of interest.

## Footnotes

1 Home – International Parkour Federation, https://internationalparkour federation.com

2 Parkour – Wikipedia, https://en.wikipedia.org/wiki/Parkour – Practice
